# Bioavailable human metabolites of a keratin-derived hydrolysate promote primary human dermal fibroblast activities and protect against oxidative stress-related damages

**DOI:** 10.3389/fnut.2026.1812320

**Published:** 2026-05-14

**Authors:** Fabien Wauquier, Line Boutin-Wittrant, Jean-Philippe Soulard, Régine Minet Quinard, Vincent Sapin, Véronique Roux, Nicolas Macian, Gisèle Pickering, Yohann Wittrant

**Affiliations:** 1 Clinic’n’Cell SAS, Faculty of Medicine and Pharmacy, Clermont-Ferrand, France; 2 BCF Life Sciences, Pleucadeuc, France; 3 Service de Biochimie et Génétique Moléculaire, CHU Gabriel Montpied, Clermont-Ferrand, France; 4 CIC INSERM 1405/Plateforme d’Investigation Clinique CHU Gabriel Montpied, Clermont-Ferrand, France; 5 INRAE, UMR 1019, UNH, Clermont-Ferrand, France; 6 Clermont Auvergne University, UMR1019 of Human Nutrition, Clermont-Ferrand, France

**Keywords:** bioavailability, clinical trial, keratin hydrolysate, oxidative stress, primary human dermal fibroblasts, skin health

## Abstract

**Introduction:**

Nutricosmetic strategies may support skin homeostasis by modulating dermal fibroblast function. Poultry feather keratin hydrolysates are a sustainable source of amino acids and low–molecular weight peptides. We investigated whether bioavailable human metabolites generated after ingestion of a keratin-derived hydrolysate (Kera‑Diet®) influence primary human dermal fibroblasts and protect against oxidative stress–related damage.

**Methods:**

Ten healthy fasted men ingested 5,000 mg Kera‑Diet®. Plasma amino acids were quantified over 240 min (UPLC–MS) to identify the absorption peak and to collect paired naïve human serum (NHS, pre-ingestion) and enriched human serum (EHS, peak time). Fibroblasts were incubated with NHS or EHS to assess viability, migration (scratch assay), and hydration markers (glycosaminoglycans and hyaluronan). Oxidative stress was induced with H₂O₂ and endpoints included ROS (DCF‑DA), apoptosis (caspase‑3), ER stress (XBP1 mRNA), and senescence (β‑galactosidase).

**Results:**

Circulating amino acids peaked at ~20 min post‑ingestion. Compared with NHS, EHS increased glycosaminoglycans (+11%) and hyaluronan release (+43%) and enhanced early migration. Under oxidative stress, Kera‑Diet® (in vitro) and EHS (ex vivo) mitigated cell stress by reducing ROS, caspase‑3 activity, XBP1 induction, and senescence, while preserving viability.

**Discussion:**

Bioavailable metabolites produced after oral keratin hydrolysate intake promote fibroblast anabolic/repair functions and blunt oxidative stress–linked ER stress, senescence, and apoptosis, supporting keratin hydrolysate–based nutritional approaches for skin health.

## 1 Introduction

Aging is associated with an increased prevalence of chronic diseases, largely driven by a progressive decline in tissue functional capacity. The skin serves as the primary biological barrier interfacing with the external environment and plays a key role in sensory perception and defence against physical, chemical, and biological aggressors (1, 2). Along with the kidneys, skin is also critical for the regulation of both, electrolytes and body fluid homeostasis (3–5). Chronic exposure to ultraviolet radiation (UV-B), referred to as photoaging, induces skin damage through the generation of oxidative stress in the dermis (6). This continuous exposure to environmental stressors alters both its appearance and functions (7).

Altered skin function is characterized by increased dryness and fragmentation of the dermal extracellular matrix. These changes primarily result from dysregulated dermal fibroblast activity (8). This manifests as a reduction in both the quantity and quality of proteins and glycosaminoglycans that constitute the extracellular matrix (9). Notably, the skin’s diminished ability to retain water is mainly attributed to the decreased synthesis of hyaluronic acid (HA) (10). Consequently, skin hydration has become a major focus of nutricosmetic strategies. Hyaluronic acid has been reported to facilitate collagen and elastin interaction and therefore to promote a proper tissue matrix configuration. In contrast, during aging, loss of hyaluronic acid may contribute to the disorganization of collagen and elastin fibers (11). Along with dryness, both a progressive accumulation of damaged proteins in the endoplasmic reticulum of skin cells (12) and an increased prevalence of senescent cells are commonly observed, further impacting skin tissue homeostasis (6).

Given the central role of the skin as an interface with the environment, cosmetic products have evolved beyond aesthetic objectives and products designed to support skin health now drive innovation across pharmaceutical, cosmetic, and food industries (13). The terms “cosmeceutical” and “nutricosmetic” have therefore been introduced to emphasize this connection and to support the concept of “beauty from within” (14).

Amino acids are the building blocks of all proteins, including the most abundant fibrous proteins in the skin, as keratins, collagen and elastin. They’ve been reported to support, wound healing, acid–base balance, water retention, protection against sunlight damage and skin microbiome (15). In addition, amino-acids and short peptides present in protein hydrolysates may be endowed with cell signalling properties that should be further considered in the design of innovative products (16, 17).

Using a well acknowledged *ex vivo* clinical approach that accounts for physiological digestive processes, we investigated a poultry feather keratin hydrolysate used as a food supplement (Kera-Diet^®^) to evaluate its nutricosmetic potential. In this context, we examined whether and how the bioavailable human metabolites resulting from the intake of this food supplement may influence the role of primary human dermal fibroblasts in skin hydration and remodelling.

## 2 Materials and methods

### 2.1 Ethics clinical trial

The study was conducted in accordance with the Declaration of Helsinki (1975, https://www.wma.net/what-we-do/medical-ethics/declaration-of-helsinki; revised in 2013). The human study was approved by the French Ethical Committee (N° SI RIPH: 24.02570.000316 / N° EudraCT/ID RCB: 2024-A01011-46/Comité de Protection des Personnes CPP Nord ouest IV; approved September 9, 2024; NCT06612866). The volunteers were informed of the objectives and the potential risks of the present study and provided their written informed consent before they participated in the study.

### 2.2 Study product

Kera-Diet^®^ consists of a hydrolysate of poultry feather keratin. It is produced by BCF Life Sciences (France) using an industrial process covered by a European patent (EP 3675811B1). In this study, the ingredient was orally administered as a powder diluted in 200 mL of water, corresponding to 5,000 mg of Kera-Diet^®^ per phase. The product formulation complies with the requirements of European regulations concerning food supplements (European Directive No. 2002/46/EC and French Decree No. 2006–352 of March 20, 2006, which transposes it). The keratin poultry feather hydrolysate (Kera-Diet^®^) is an active food ingredient. It presents a unique profile of 17 amino acids dispatched either in free form (>94%) and small peptides of low molecular weight (<6%; all peptides being <800 Da).

### 2.3 Human study design and pharmacokinetic of absorption

A cohort of ten healthy male volunteers (mean age: 26.2 ± 3.9 years; BMI: 23.94 ± 1.7 kg/m^2^; body weight >60 kg; not undergoing any pharmacological treatment; ethnicity not specified) participated in this study. All subjects were screened to confirm normal hematological parameters, as well as renal (urea and creatinine) and hepatic function, including aspartate aminotransferase (AST), alanine aminotransferase (ALT), and gamma-glutamyltransferase (GGT) activities. Blood samples were collected from each participant using serum-separating tubes. The resulting serum was aliquoted and stored at the Centre d’Investigation Clinique de Clermont-Ferrand—Inserm 1,405, a specialized research facility certified according to the French standard NF S 96900, ensuring sample quality and compliance with regulatory and ethical requirements.

The initial phase of this study was designed to determine the absorption peak of Kera-Diet^®^ metabolites. Ten healthy volunteers, who had fasted for a minimum of 12 h, received an oral dose of 5,000 mg of Kera-Diet^®^. The dosage was established based on previously validated preclinical and clinical studies (16, 18–22). Venous blood samples (approximately 9 mL) were collected from the median cubital vein prior to ingestion and subsequently at 5, 10, 20, 30, 45, 60, 80, 100, 120, 140, 180, and 240 min post-ingestion. Serum was isolated from these samples and stored at −80 °C until analysis. The absorption profile of Kera-Diet^®^ was assessed by monitoring the blood concentrations of amino acids throughout the kinetic study.

Upon determination of the absorption peak, the volunteers were recalled for collection of the enriched serum fraction. In this second clinical phase, after a 12-h fast, the same ten healthy volunteers, were administered 5,000 mg of Kera-Diet^®^. Approximately 48 mL of venous blood was drawn from the cubital vein prior to ingestion to obtain the naïve serum fraction (naive human serum, NHS). At the time corresponding to the maximum absorption peak, an additional 48 mL of blood was collected to obtain the enriched serum fraction (enriched human serum, EHS), which contained the circulating bioavailable metabolites resulting from Kera-Diet^®^ absorption. All serum samples were stored at −80 °C until further analysis.

### 2.4 Determination of amino-acid concentrations in blood samples by UPLC–MS

Chemicals and reagents: Liquid Chromatography Mass Spectrometry (LC–MS) grade acetonitrile (ACN) and formic acid were purchased from Carlo Erba (Lyon, France). Deionized water (18.2 MΩ) was obtained using a Milli-Q^®^ IQ 7003 water purification system (Sigma-Aldrich, Lyon, France). The Kairos™ amino acid kit, including calibrators, internal standards, quality controls, and reagents, was purchased from Waters (Guyancourt, France). Sample preparation: Plasma samples were centrifuged for 10 min at 2,500 g, aliquoted, and stored frozen at −80 °C until analysis. The samples were prepared using the Waters™ Kairos™ amino acid kit, which enables the simultaneous analysis of 24 biologically relevant amino acids. For each sample, 50 μL of plasma was dispensed directly into a 0.5 mL Eppendorf tube, to which 50 μL of internal standard precipitation solution was added. This solution contains stable-isotope-labeled (SIL) internal standards in 10% (v/v) sulphosalicylic acid. After thorough mixing, the samples were diluted with 50 μL of H₂O and centrifuged for 15 min at 9000 g. Subsequently, 10 μL of the supernatant was transferred into a conical-bottom glass HPLC vial preloaded with 70 μL of borate buffer. The mixture was thoroughly mixed, and 20 μL of derivatization solution (AccQ-Tag Ultra reagent) was added. The sample was incubated for 10 min at 55 °C. Calibration standards and quality controls (QCs) were processed using the same protocol. Six calibration points were constructed, with concentration ranges of 5–1,000 μM (2.5–500 μM for cysteine). Liquid chromatography and mass spectrometry: The chromatographic system consisted of an Acquity UPLC I-Class system (Waters, Guyancourt, France) equipped with a Sample Manager—Flow Through Needle (SM-FTN), a Binary Solvent Manager (BSM), and a Column Heater (CH). The mass spectrometer used was a triple quadrupole Xevo TQ-S micro (Waters, Guyancourt, France) equipped with an Electrospray Ionization (ESI) source. The mass spectrometer was operated in positive ion mode using electrospray ionization with the following optimized parameters: CCHillary voltage: 2 kV; source temperature: 150 °C; desolvation temperature: 500 °C; desolvation gas flow: 1000 L/h; cone gas flow: 20 L/h. Analyses were performed in scheduled multiple reaction monitoring mode (MRM). A unit resolution was used for both Q1 and Q3 quadrupoles. Data acquisition and processing were performed using MassLynx 4.2 and TargetLynx XS 4.2 (Waters), respectively. The quantification limits ranged from 0.2 to 10 μM. Two microliters of processed sample solution were injected into the LC system. A CORTECS UPLC C18 column (150 × 2.1 mm, 1.6 μm, Waters, Guyancourt, France) was used for chromatographic separation. The column was maintained at 55 °C. The mobile phase consisted of solvent A (H₂O + 0.1% formic acid, v/v) and solvent B (ACN + 0.1% formic acid, v/v). The elution pump was programmed as follows: 1% B (0.5 mL/min) for 1-min, linear gradient to 13% B over 4 min, linear gradient to 15% B over 4.5 min, linear gradient to 95% B in 1 min, 99% B for 1 min, linear gradient to 25% B in 0.1 min, and equilibration for 1.4 min. The total chromatographic run time was 12 min.

### 2.5 Human primary dermal fibroblasts (HDFs) cultures

Human primary dermal fibroblasts derived from an adult donor were obtained from Sigma-Aldrich (Lyon, France, 106-05A). Cells were maintained in Dulbecco’s Modified Eagle Medium (DMEM, Biowest, L0066-500) supplemented with 10% fetal calf serum (FCS, Invitrogen) and 1% penicillin/streptomycin (Life Technologies, Villebon-Sur-Yvette, France). Cultures were incubated at 37 °C in a humidified atmosphere containing 5% CO₂ and 95% air.

**Figure 1:**
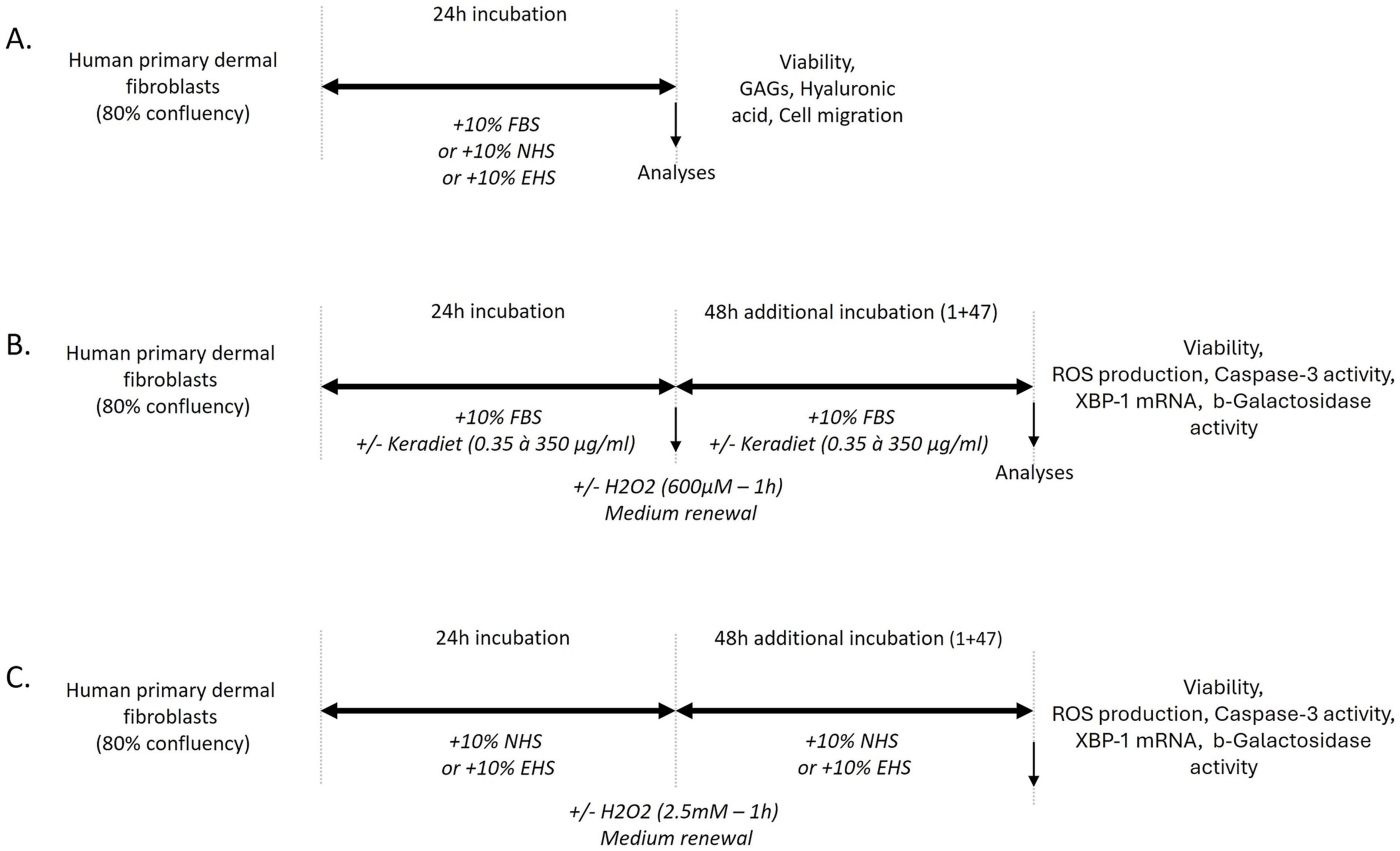
Experimental designs. **(A)** *Ex vivo* procedures in the absence of an oxidative stress. **(B)** *In vitro* investigations in the presence of an oxidative stress. **(C)** *Ex vivo* cultures under oxidative stress conditions.

To investigate the effects of Kera-Diet^®^, three experimental protocols were employed. The first protocol assessed the impact of Kera-Diet^®^ under non-stress conditions (Figure 1A), with cells cultured in the presence of either naive human serum (NHS) or enriched human serum (EHS). The second protocol evaluated the dose-dependent benefits of Kera-Diet^®^ under oxidative stress using standard foetal bovine serum (Figure 1B). The third protocol examined the influence of circulating metabolites using human serum, following the Clinic’n’Cell methodology (DIRV INRA 18–00058), comparing naive and enriched serum under oxidative stress (Figure 1C). Human sera were diluted ten times to match the quantity ingested (5,000 mg) and the recommended daily intake of Kera-Diet^®^ (500 mg). Human serum samples were not pooled (*n* = 10), and all assays were performed in three to six replicates.

Briefly, cells were seeded at a density of 15,000 cells/cm^2^ in either 96- or 24-well plates, with 100 μL or 500 μL of culture medium, respectively. Cells were allowed to proliferate for 3 days in maintenance medium until reaching 80% confluency, after which they were subjected to the respective experimental protocols as outlined in Figures 1A–C.

### 2.6 Cell viability

The *ex vivo* cell viability was determined using an XTT-based method (Cell Proliferation Kit II, Sigma-Aldrich) according to the supplier’s recommendations. Optical density was measured at 450 nm.

### 2.7 Cell migration assay

After 24 h of pre-treatment, monolayers of dermal fibroblasts were subjected to a linear scratch in the middle of the well using a P1000-tip. Then, culture media were changed to removed floating cells and scratched monolayers were placed under a video microscope allowing timelapse investigations at 37 °C and 5%CO2/95% air. X, Y, and Z coordinates were set for each well to follow the migration of the primary HDFs covering the scratched area on 24 h. Surface covered by cells was quantified using ImageJ software 1.53 and calculated as a percentage (ratio) of the initial wounded surface to avoid bias in comparison.

### 2.8 Glycosaminoglycans (GAGS) assay

A dimethylmethylene blue (DMB) assay was used to detect GAG production in cell lysates as previously described (23). DMB solution was prepared at a final concentration of 46 mmol/L in a pH 3 adjusted buffer: 40 mmol/L NaCl, 40 mmol/L glycine. Sample concentrations were determined by mixing 25 μL of cell extract with 200 μL of DMB reagent. Following 30 min incubation, the absorbance was read at 595 nm on an ELX808 IU spectrophotometer (BioTek Instruments, Winooski, VT, USA). The GAG content was determined using a standard curve of chondroitin sulfate (Sigma). Results are expressed as μg of GAG per μg of total proteins determined by BCA assay (Sigma). For human serum, measurements were performed in quadruplicates for each sample of the ten volunteers.

### 2.9 Hyaluronan quantification

In HDFs, Hyaluronan levels were evaluated in cell culture supernatant using Hyaluronan Quantikine ELISA Kit (DHYAL0) according to the manufacturer’s recommendations. For human serum, measurements were performed in quadruplicates for each sample of the ten volunteers.

### 2.10 ROS production/DCF-DA staining

Human primary dermal fibroblasts were seeded on a 96-well dark-wall clear-bottom plate at a density of 12,000 cells/cm^2^. Twenty-four hours after palmitate (PA) stimulation, cells were washed and incubated with 5 μM of 2′,7′-dichlorofluorescin diacetate (DCF-DA) solution (ab113851, Abcam) for 45 min at 37 °C in the dark, then rinsed with the dilution buffer according to the manufacturer’s protocol. Fluorescence was measured using a fluorescence plate reader (Berthold − Mitras) at Ex/Em = 485/535 nm in end-point mode.

### 2.11 Caspase-3 activity

Caspase-3 activity in cells was evaluated using the Caspase-3 Assay Kit (Abcam, Paris, France—ab39401) according to the manufacturer’s protocol. To lyse the frozen monolayer of human primary dermal fibroblasts, cells grown on a twelve-well plate, 200 μL of the provided lysis buffer was used per well. Fifty microliters of freshly prepared lysate were then mixed with 55 μL of reconstituted reaction mix. Optical density at 405 nm was measured every 2 min for 90 min at 37 °C.

### 2.12 ß-galactosidase activity

ß-Galactosidase activity was evaluated using a staining kit from Abcam (Paris, France, ab65351) according to manufacturer’s protocol. Following the 24 h treatment with H_2_O_2_, cells were washed once with PBS before the “Fixative Solution” from the kit was applied for 15 min at room temperature. Then, after two more PBS washes, fixed cells were covered with the “Staining solution mix” and incubated overnight at 37 °C. Development of the blue color within the cells was analysed the following day by observation under a microscope. Relative staining units were evaluated using ImageJ software.

### 2.13 Real-time RT-qPCR

mRNA from HDFs were isolated using TRIzol™ Reagent (Ambion – Life Technol-ogies) according to the supplier’s recommendations. The expression level of XBP-1 mRNA was measured by RT-qPCR (PowerUp SYBRgreen, Applied Biosystems). β-Actin was used as a housekeeping gene. Primers were designed as follows: XBP1-F: 5′- CGC TGT CTT AAC TCC TGG TTC -3′; XBP1-R: 5′- CTG GAA CAG CAA GTG GTA GA − 3′; ACTβ-F: 5′-ATT GGC AAT GAG CGG TTC-3′; ACTβ-R: 5′-GGA TGC CAC AGG ACT CCA-3′.

### 2.14 Cell lysis

The lysis buffer was prepared by mixing 50 mmol/L Tris pH 7.8, 150 mmol/L NaCl, 0.5% sodium deoxycholate, and 1% NP40. Cell lysates were stored at −80 °C until analysis.

### 2.15 Protein quantification

Protein content was measured using the BCA Protein Assay Kit (Sigma-Aldrich, Saint-Quentin-Fallavier, France). The BCA protein assay is based on a biuret reaction, where the reduction of Cu2 + to Cu + in the presence of proteins in an alkaline environment is proportional to the protein concentration. The chromogenic reagent bicinchoninic acid chelates the reduced copper, forming a purple complex that absorbs at 562 nm.

### 2.16 Statistics

**Figure 2:**
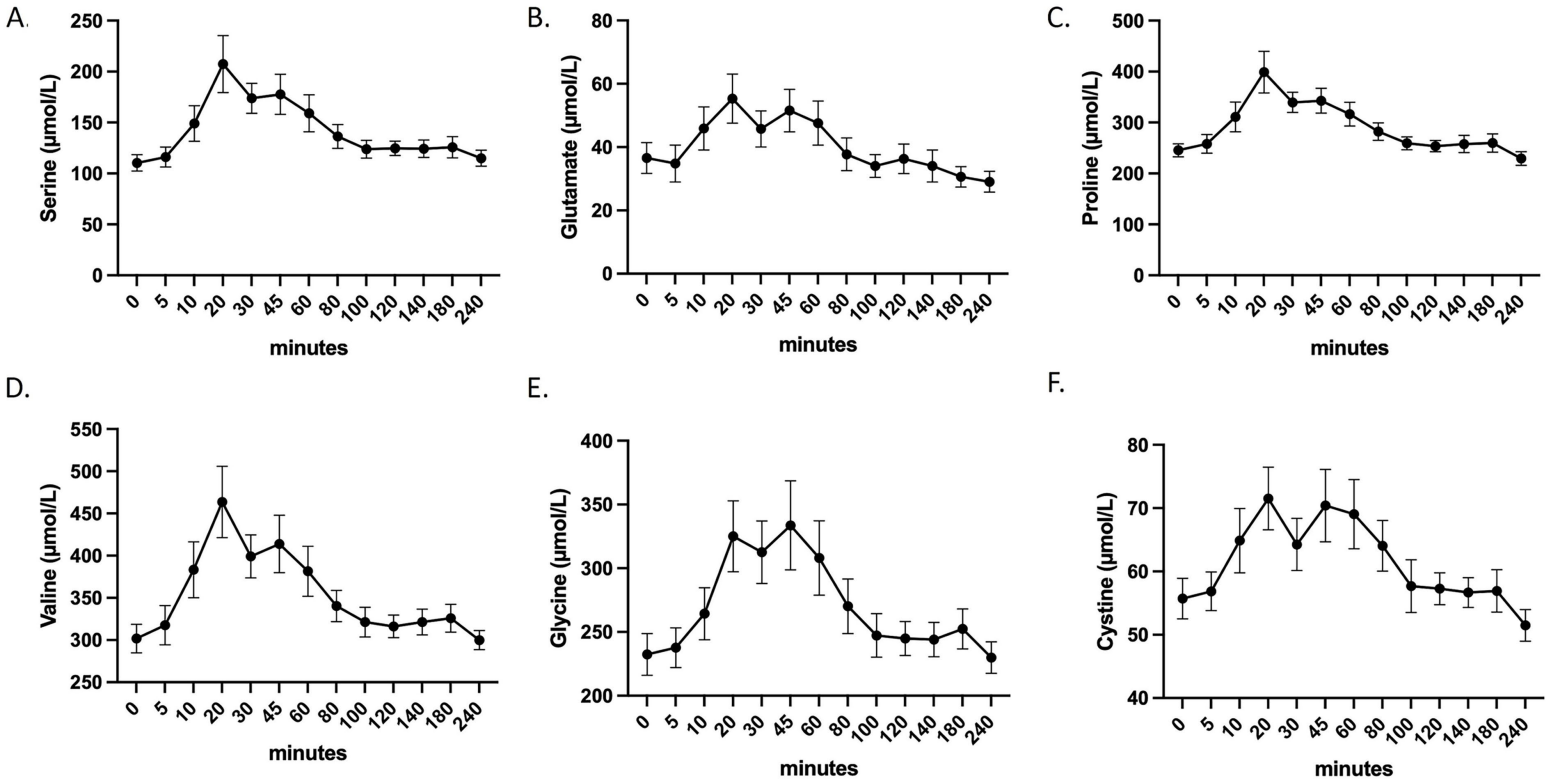
Evolution of the blood concentration of the six most representative amino-acids composing Kera-Diet^®^. **(A)** Serine. **(B)** Glutamate. **(C)** Proline. **(D)** Valine. **(E)** Glycine. **(F)** Cystine.

**Figure 3:**
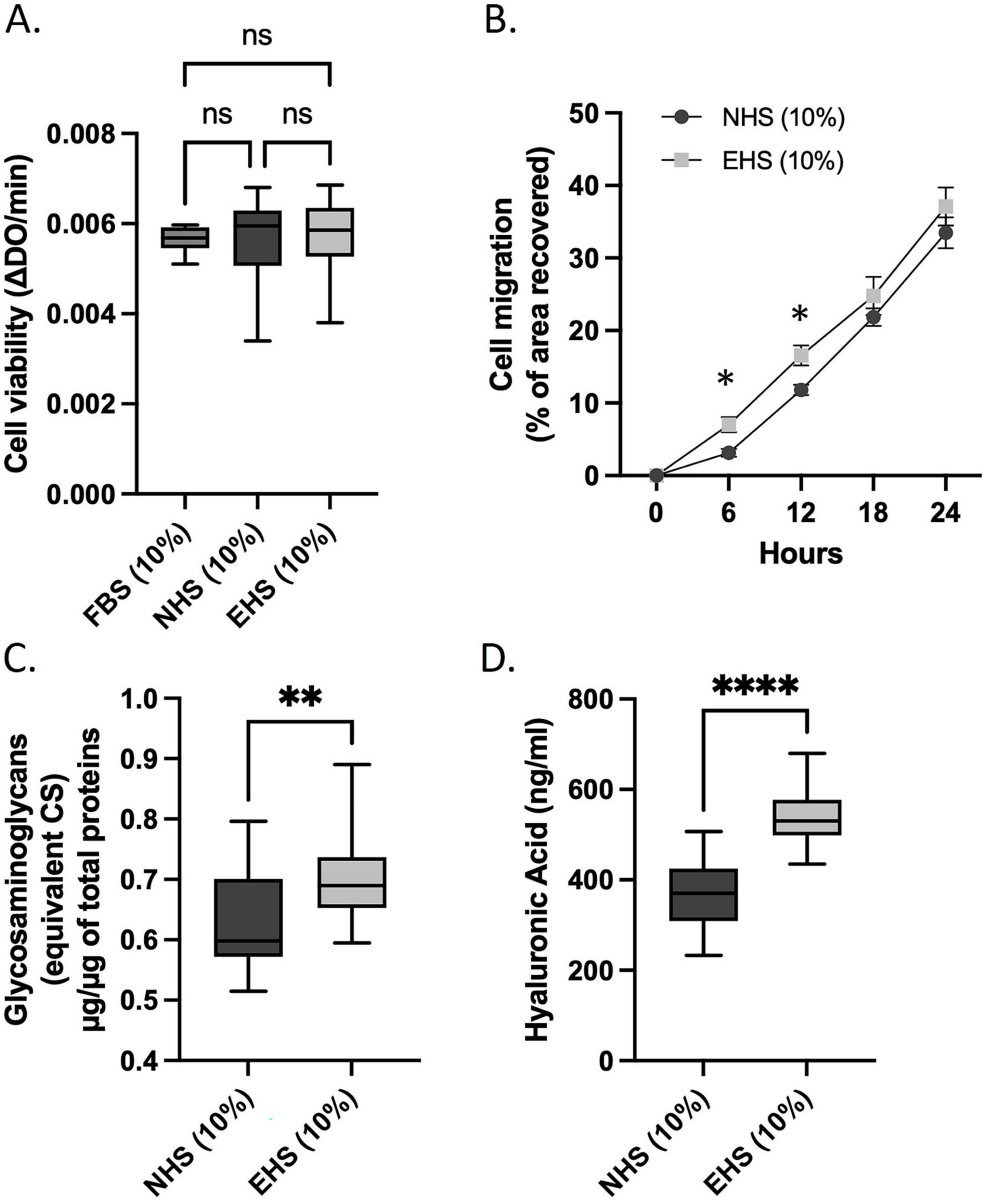
Primary human dermal fibroblasts (adult) subjected to *ex vivo* procedures. Comparison of cell viability incubated either with foetal bovine serum (FBS), naïve human serum (NHS) or enriched human serum (EHS) as evaluated by a XTT-based assay **(A)**. Evaluation of cell migration properties **(B)**. Determination of both glycosaminoglycans (GAGs) and hyaluronic acid synthesis **(C,D)**. Measures were performed in quadruplicate (biological replicates, *n* = 4, except for viability, *n* = 6) per condition and per volunteer (*n* = 10 volunteers). **A,C, D** Boxes indicate median and interquartile range (lower and upper), while whiskers indicate minimum and maximum. **B**: Plots represent the mean ±SD. For each panel: *: *p* < 0.05; **: *p* < 0.01; ****: *p* < 0.0001; ns: *p* > 0.05.

**Figure 4:**
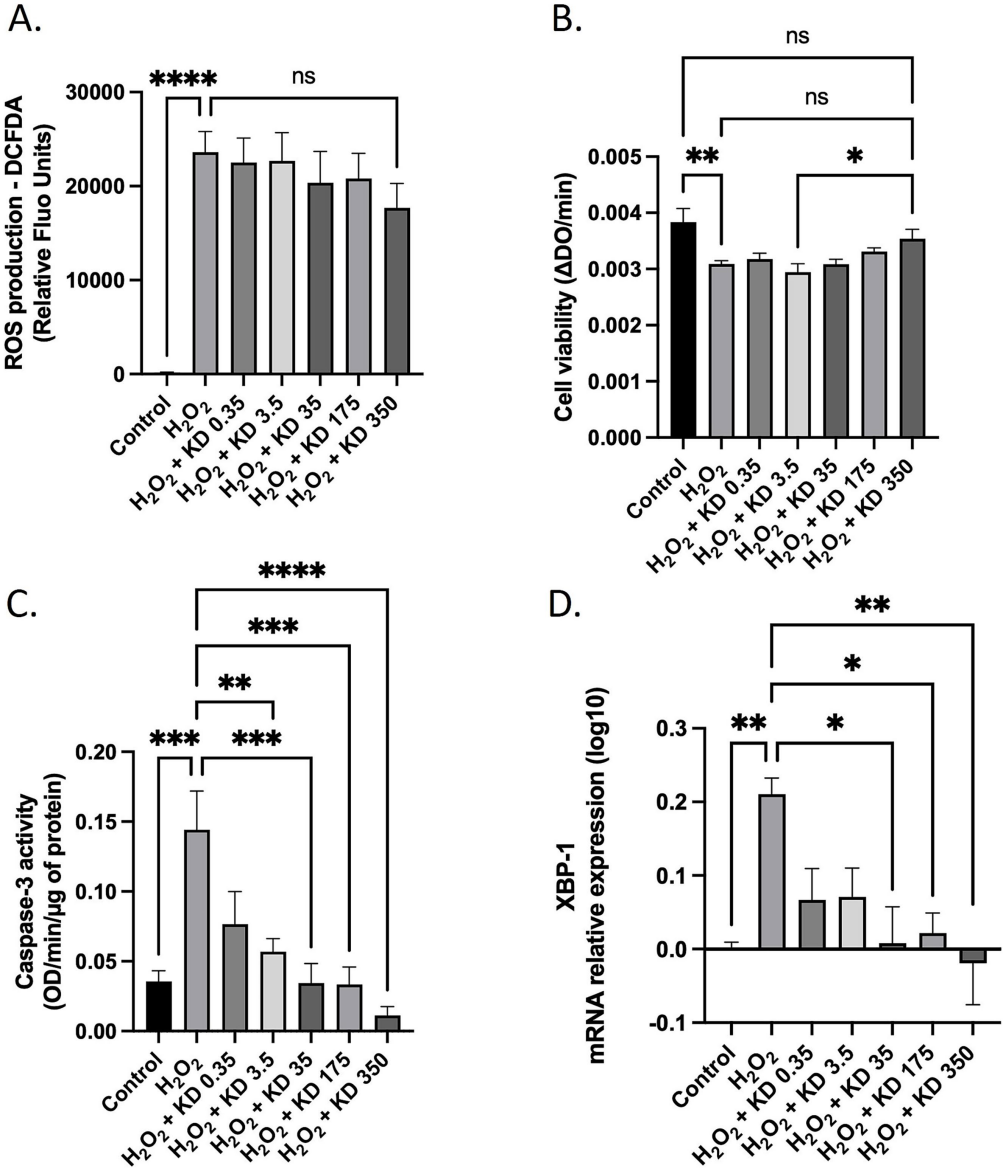
Primary human dermal fibroblasts (adult) subjected to oxidative stress *in vitro*. Cells were incubated with foetal bovine serum (FBS 10%) with or without H2O2 with increasing doses of KD (Kera-Diet^®^) (0.35 to 350 mg/L of culture media). ROS production assayed by DCFDA probe **(A)**. XTT-based evaluation of cell viability **(B)**. Caspase-3 activity **(C)**. XBP-1 relative mRNA expression **(D)**. Measures were performed in quadruplicate (biological replicates, *n* = 4, except for viability, *n* = 6) per condition and per volunteer (*n* = 10 volunteers). Histograms represent the mean ±SD. For each panel: *p < 0.05; **p < 0.01; ***p < 0.001; ****p < 0.0001; ns: *p* > 0.05.

**Figure 5:**
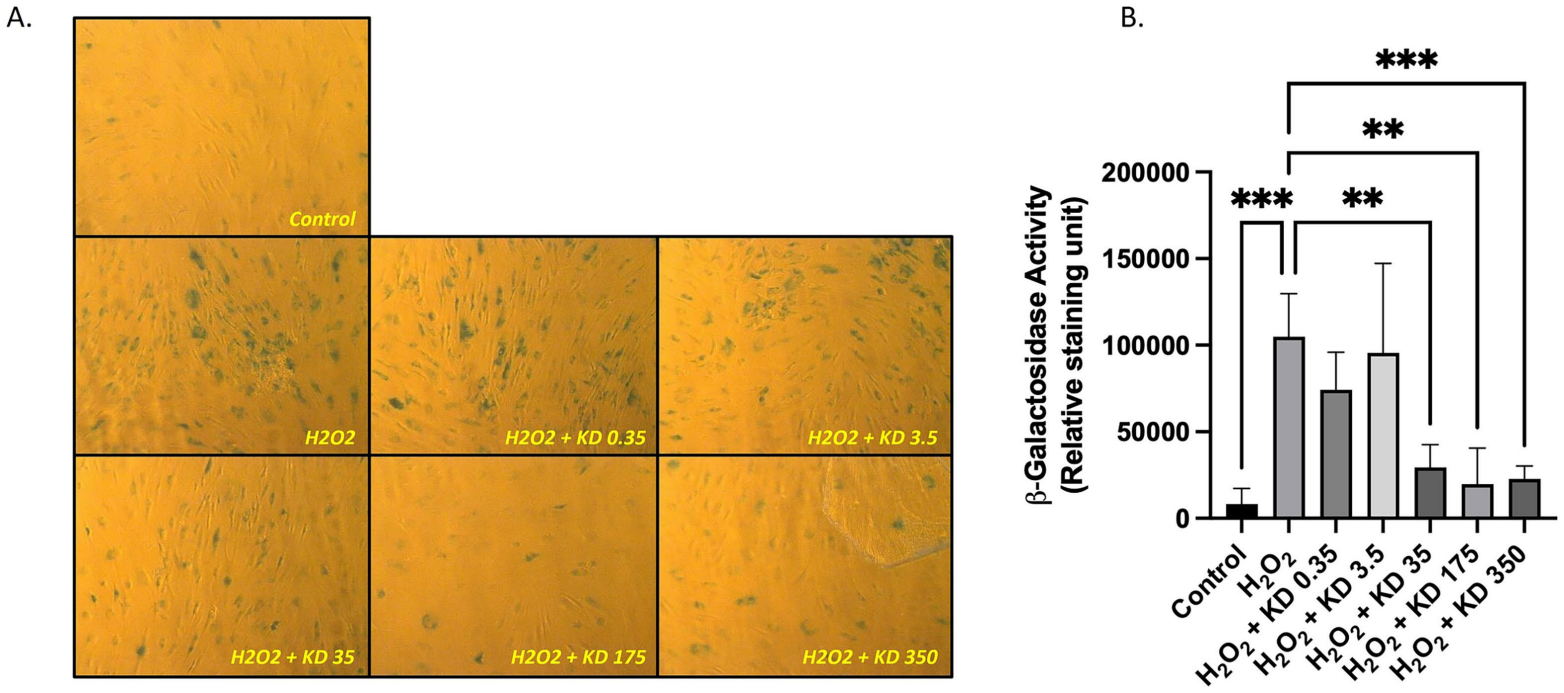
Primary human dermal fibroblasts (adult) subjected to oxidative stress *in vitro*. Cells were incubated with foetal bovine serum (FBS 10%) with or without H2O2 with increasing doses of KD (Kera-Diet^®^) (0.35 to 350 mg/L of culture media). *β*-Galactosidase staining **(A)**. Quantification of β-Galactosidase staining **(B)**. Measures were performed in triplicate (biological replicates, *n* = 3) per condition and per volunteer (*n* = 10 volunteers). Histograms represent the mean ±SD. **: *p* < 0.01; ***: *p* < 0.001.

**Figure 6:**
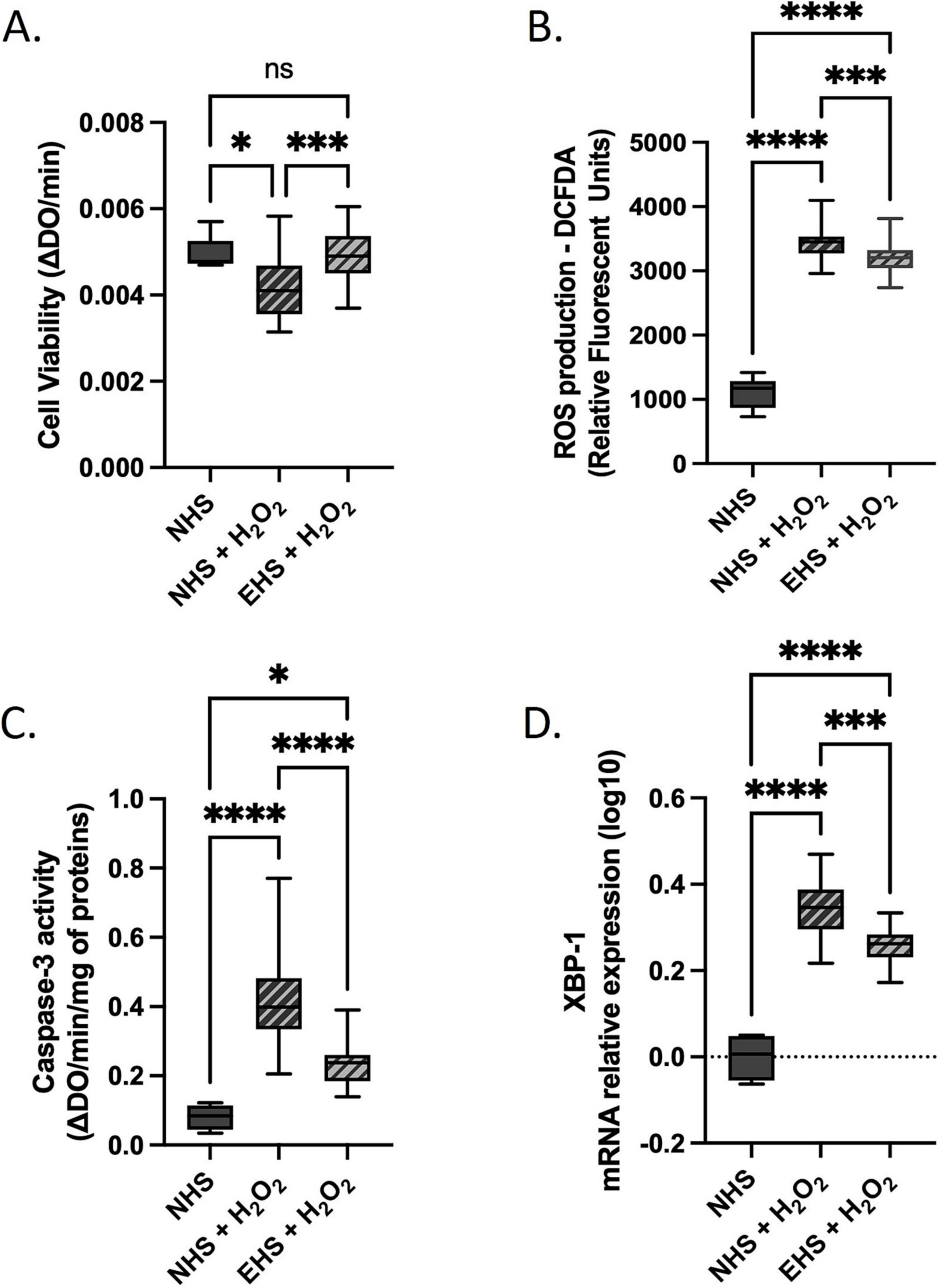
Primary human dermal fibroblasts (adult) subjected to oxidative stress *ex vivo*. Cells were incubated either naïve human serum (NHS) or enriched human serum (EHS – containing Kera-Diet^®^ metabolites) with or without H_2_O_2_. **(A)** XTT-based evaluation of cell viability. **(B)** ROS production assayed by DCFDA probe. **(C)** Caspase-3 activity. **(D)** XBP-1 relative mRN A expression (D). Measures were performed in quadruplicate (biological replicates, *n* = 4, except for viability, *n* = 6) per condition and per volunteer (*n* = 10 volunteers). Boxes indicate median and interquartile range (lower and upper), while whiskers indicate minimum and maximum. *: *p* < 0.05; **: *p* < 0.01; ***: *p* < 0.001; ****: *p* < 0.0001; ns: *p* > 0.05.

Statistical analyses and figure generation were performed using Prism V.10.4.1 (GraphPad Software). The statistical plan included a Shapiro–Wilk normality test to determine if the data were consistent with Gaussian distribution. If the data were not normally distributed, a Kruskal–Wallis non-parametric test was used, followed by Dunn’s test for *post hoc* comparisons. For normally distributed data with equal variance, one-way or two-way ANOVA with Tukey’s test for multiple comparisons was applied. For migration assays, a two-way ANOVA was applied followed by Tukey’s post hoc test. For Figures 2, 3B, 4, 5, Supplementary Figure S1, S2, values are presented as the means ± SD. For Figures 3, 6, and Supplementary Figure S3, values are presented as box plots indicating median and interquartile range (lower and upper), with whiskers indicating minimum and maximum values. Statistical significance is indicated as follows: * for *p* < 0.05; ** for *p* < 0.01; **** for *p* < 0.0001; ns for *p* > 0.05.

## 3 Results

### 3.1 Absorption profile of circulating amino-acids detected in human serum following Kera-Diet^®^ ingestion

Kera-Diet^®^ was ingested and digested by fasted healthy volunteers, and the concentration of circulating amino-acids in the bloodstream was monitored over time using a kinetic approach to determine their absorption profile on a 240 min period of time. A total of 24 amino-acids were monitored but only the six most representative of Kera-Diet^®^ ingredient are presented in Figure 2 (see Supplementary Table S1 and Supplementary Figures S1, S2 for Kera-Diet^®^ amino acid profile). UPLC-MS analyses show that following Kera-Diet^®^ ingestion, the concentration of these amino-acids rapidly increased to reach a maximum at 20 min for glutamate (36.58 μmol/L (t = 0 min) to 55.33 μmol/L (t = 20 min)), proline (245.45 μmol/L (t = 0 min) to 399.1 μmol/L (t = 20 min)), valine (301.75 μmol/L (t = 0 min) to 463.69 μmol/L (t = 20 min)) and serine (110.33 μmol/L (t = 0 min) to 207.42 μmol/L (t = 20 min)), while glycine peaks between 20 and 45 min (232.38 μmol/L (t = 0 min) to 325.02 μmol/L (t = 20 min) and 333.65 μmol/L (t = 45 min)). All 17 amino-acids composing Kera-Diet*^®^* were found in the blood samples (Supplementary Figure S2). According to the consistency of the absorption profiles, collection of the enriched serum for the second phase of the clinical protocol was set at 20 min post-ingestion.

### 3.2 Validation of the cell model for *ex vivo* procedures

The main objective of this *ex vivo* investigation was to evaluate the impact of human serum enriched with amino-acids and short peptides resulting from Kera-Diet*^®^* ingestion on primary human dermal fibroblasts. To ensure the physiological relevance of our *ex vivo* approach, and according to culture settings shown in Figure 1A, we checked the influence of the different human sera versus regular fetal bovine serum on cell growth using a XTT-based method (Figure 3A). Neither naïve nor enriched human serum processed according to the Clinic’n’Cell methodology (DIRV#18–0058; see the Patents section) exerted any adverse effects on cell viability as compared to conventional foetal bovine serum and support further investigations.

### 3.3 Human serum following Kera-Diet^®^ ingestion promotes both migration and hydration in primary human dermal fibroblasts in the absence of stress

Skin hydration and healing are required for the maintenance of skin function. Glycosaminoglycans (GAGs) and hyaluronic acid are key contributors for skin hydration therefore we checked their production in primary human dermal fibroblasts. Incubation with human serum containing Kera-Diet^®^ circulating metabolites (EHS) significantly enhanced both GAGs and hyaluronic acid synthesis and release as compared to naïve serum (NHS), +11% EHS vs. NHS and +43% EHS vs. NHS for GAGs and hyaluronic acid, respectively, (Figures 3C,D). A similar trend was observed in primary human keratinocytes (Supplementary Figure S3). Notably, chondroitin sulfate synthase expression in dermal fibroblasts was upregulated by the presence of the metabolites (Supplementary Table S2). Moreover, the presence of Kera-Diet^®^ metabolites promoted cell migration at all timepoints, but significance was only observed for early timepoints (6 h; +44.7% EHS vs. NHS and 12 h; +40.3% EHS vs. NHS) (Figure 3B).

### 3.4 Kera-Diet^®^ limits oxidative stress *in vitro*

Then we hypothesized that Kera-Diet^®^ may exert skin protective properties by limiting oxidative stress damages on primary human dermal fibroblasts. We checked this hypothesis *in vitro*. Cells were exposed to H_2_O_2_ in the presence of increasing dose of Kera-Diet^®^ (raw material, not human metabolites). In this design, the serum used was from bovine origin to meet regular culture settings (Figure 1B). We first validated the model of stress. As observed on Figure 4A, the incubation with H_2_O_2_ massively increased ROS production (x123 fold over control condition) supporting further investigation. The presence of Kera-Diet^®^ tended to limit this rise (x92 fold over control condition; −25% H_2_O_2_ + KD350 vs. H_2_O_2_). However, the trends remained insignificant (Figure 4A). Consistent with this observation, H_2_O_2_ significantly altered cell viability (−19.5% H_2_O_2_ vs. control—Figure 4B). Once again, the incubation with increasing doses of Kera-Diet^®^ tended to restore this parameter but without reaching a significant threshold yet, and H_2_O_2_ and H_2_O_2_ + KD350 groups remained insignificantly different. Still, the same observation was made when comparing the groups H_2_O_2_ + KD350 and control supporting both the trend of the restoration and the rationale for a higher dose (−7.8% H_2_O_2_ + KD350 vs. control—Figure 4B). Accordingly, H_2_O_2_ significantly triggered apoptosis mechanisms and significantly promoted caspase-3 activity (+311% H_2_O_2_ vs. control—Figure 4C). Incubation with Kera-Die^t®^ remarkably abolished H_2_O_2_-induced caspase-3 activity in a dose-dependent manner. The statistical significance of the inhibition was reach with concentration as low as 3.5 mg/L. Total inhibition was obtained from the dose 35 mg/L.

### 3.5 Kera-Diet^®^ protects against oxidative stress-induced ER-stress and senescence

At crossroads between cell viability and oxidative stress, ER-stress and cellular senescence are major player in skin homeostasis. As expected, the incubation with H_2_O_2_ up-regulated the expression of XBP-1, a cornerstone in ER-stress pathway (+62% H_2_O_2_ vs. control condition—Figure 4D). Incubation with Kera-Diet^®^ fully inhibited H_2_O_2_-induced XBP-1 expression in a dose-dependent manner. The statistical significance of the inhibition was reached from 35 mg/L and so was the total inhibition. Regarding senescence, H_2_O_2_ stimulated β-Galactosidase activity (x12.7 fold over control condition—Figures 5A,B) a classical marker of cell senescence. The presence of Kera-Diet^®^ significantly lessened the H_2_O_2_-induced β-Galactosidase activity (x4.6 fold over control condition; −64% H_2_O_2_ + KD350 vs. H_2_O_2_) in a seemingly dose dependent fashion.

### 3.6 Kera-Diet^®^ lowers oxidative stress hallmarks *ex vivo*

To reach a more physiological point of view, oxidative stress-related investigations were also performed *ex vivo* and primary human dermal fibroblasts were incubated either with naïve human serum (NHS) or enriched human serum (EHS – containing Kera-Diet^®^ metabolites) in the presence or absence of H_2_O_2_ stimulation. To reach a significant oxidative stress in such an experimental design (Figure 1C), H_2_O_2_ concentration was increased from 600 μM to 2.5 mM. We speculate that this difference may rely on the serum properties (bovine vs. human) but we did not demonstrate it. Anyway, consistent with the *in vitro* data, H_2_O_2_ significantly decreased cell viability (−16.5% NHS + H_2_O_2_ vs. NHS—Figure 6A) while fostering ROS production, caspase-3 activity and XBP-1 expression, by +207%; +399% and +119%, respectively, (NHS + H_2_O_2_ vs. NHS—Figures 6B–D respectively). Remarkably, the presence of Kera-Diet® human metabolites hindered H_2_O_2_-induced stress, with a full inhibition regarding cell viability (Figure 6A), a slight but significant limitation of ROS production (+183% NHS + H_2_O_2_ vs. NHS; −11.5% EHS + H_2_O_2_ vs. NHS + H_2_O_2_—Figure 6B), a reduction of caspase-3 activity (+190% NHS + H_2_O_2_ vs. NHS; −52.4% EHS + H_2_O_2_ vs. NHS + H_2_O_2_—Figure 6C) and finally a decrease of H_2_O_2_-induced XBP-1 expression (+80% NHS + H_2_O_2_ vs. NHS; −39% EHS + H_2_O_2_ vs. NHS + H_2_O_2_—Figure 6D).

## 4 Discussion

Using an *in vitro* experimental design, we report that the ingredient Kera-Diet^®^ protected against oxidative stress-related damages on primary human dermal fibroblasts. Additionally, using an integrative *ex vivo* clinical approach considering the digestive process, we confirmed that the ingredient Kera-Diet^®^ was digestible and bioavailable. The related circulating metabolites also exhibited protective properties under oxidative stress and even promoted fibroblast anabolism and migration in the absence of stress.

These findings are consistent with previously reported results. We recently conducted a randomized clinical trial with a feather-keratin hydrolysate considered as a food supplement. A panel of adult women showing aging physiological signs were given 500 mg to 1,000 mg of the hydrolysate for 90 days. As compared to placebo, the supplementation with keratin hydrolysate improved skin roughness, wrinkle features, deep skin moisturization, skin maximum elongation and elasticity, skin thickness, gloss of skin, hair and nails, and nail hardness (22). Together, these findings provide insight into the mechanism of action and further support the observed benefits on the panel. These results also consistently echo with literature data. Interestingly, soluble keratin maintained cell viability in thermally stressed dermal fibroblasts (24). Feathers-derived peptides with low molecular weight (< 3 kDa) were reported to have a potent antioxidant activity *in vitro* on gingival fibroblasts (25). In a human keratinocyte cell line (HaCat cells) feather keratin hydrolysis peptides had both the ability to scavenge H_2_O_2_-induced ROS production and promote cell migration, while remaining non-toxic (26).

In addition to the enhancement of fibroblast migration by Kera-Diet^®^, low molecular weight keratin hydrolysates were shown to improve skin wound healing (27). In this case, the size of the biopeptides may influence their bioavailability and subsequently their activity. *In vitro*, in a Caco-2 model, transport efficiency across the cell monolayer was positively correlated with a greater protein hydrolysis (collagen) (28). According to its hydrolysis process, Kera-Diet^®^ is mainly composed of free amino-acids (94–95%). However, small peptides less than 800 Da are also present in Kera-Diet^®^ which are likely to account for the observed biological activity. This point remains to be further characterized.

Skin aging is characterized by dryness and subsequent matrix networks fragmentation in the dermis. Therefore, hydration has become a major concern for nutricosmetic strategies. Hyaluronic acid has been reported to facilitate collagen and elastin interaction and therefore to promote a proper tissue matrix configuration. In contrast, during aging, loss of hyaluronic acid may contribute to the disorganization of collagen and elastin fibers (11). Collectively, the up-regulation of both glycosaminoglycans and hyaluronic acid by the human metabolites of Kera-Diet^®^ likely contribute to explain the clinical improvements observed from our previous clinical study. Along with keratin, collagen hydrolysates have been reported for their nutracosmetical potential and their ability to promote anabolism signals (20). These data potently support the idea that such protein hydrolysates are endowed with cell signalling properties and should no longer be considered as simple building blocks (16).

Known as photoaging, the chronic exposure to ultraviolet radiation (UV-B) leads to a myriad of toxic signalling events including ROS generation in dermis (6). Such an oxidative stress leads to a progressive accumulation of damaged proteins in the endoplasmic reticulum of skin cells driving the UPR (Unfolded Protein Response) (12). This signalling cascade activates IRE1, PKR-like ER kinase (PERK), and ATF6 to restore endoplasmic reticulum homeostasis (29). PERK activation induces an antioxidant response (30, 31) while XBP-1 upregulation results from the upstream activation of IRE1 (32, 33). Alternatively, when cell homeostasis cannot be restored, this oxidative stress path the way for cellular senescence and ultimately cell apoptosis (34). Kera-Diet^®^ protected against oxidative stress-induced XBP1-expression, β-Galactosidase activity and ultimately caspase-3 activity both *in vitro* in a dose dependent fashion and *ex vivo* in a more physiological perspective supporting Kera-Diet^®^ benefits on skin and providing insights on the mode of action.

β-Galactosidase activity is a classic marker of cellular senescence, a state of highly stable cell cycle arrest. β-Galactosidase activity is thought to be crucial in malignant cell transformation (35). The presence of senescent cells in the skin can alter the extracellular matrix and the function of the neighbouring cells as well, exacerbating photodamage and increasing the risk of carcinogenesis (6). These observations further support new avenues to be investigated regarding the potential health benefit of Kera-Diet^®^ in the preservation of skin integrity and the prevention of skin pathology.

This study also has limitations to be discussed. In the previous RCT we conducted, volunteers were given 500 to 1,000 mg of Kera-Diet^®^. The dose of 500 mg per day served as reference for the different protocol designs. For *in vitro* studies, doses were set according to an estimated absorption ranging from 1 to 100% with either a distribution restricted to the blood compartment or a full distribution to all the extracellular liquid volume. For the *ex vivo* clinical approach, since fibroblasts are cultured with 10% of human serum (either naïve or enriched) we considered ten times the daily dose. This single exposure of 5,000 mg is quite consistent with both, previous *ex vivo* clinical trials with similar protein hydrolysates ranging from 12 to 25 g (19–21) and other regular clinical trials run with a daily supplementation ranging from 200 mg/day to 10 g/day (36–40) and mostly related to collagen hydrolysates. Besides, although volunteers are in a fasted state supporting that serum enrichment relies only on Kera-Diet^®^ ingestion, one may speculate on an indirect modification of the serum due to a post-prandial-like state that could be consecutive to whatever ingredient consumption. So far, we have never encountered such an issue but this point it worth noting.

One may also question the use of H_2_O_2_ as a model of stress for skin cells. Photoaging results from the chronic exposure to UV-B that drives the generation of reactive oxygen species in the dermis (6). H_2_O_2_ is now acknowledged as a relevant stress to investigate senescence, endoplasmic reticulum chaperoning mechanisms impairment and unfolded protein response activation in fibroblasts and therefore has become widely adopted to screen food supplement with nutracosmeticals properties (34). For instance, a similar stress model was recently used to decipher the role of curculigosides in mitigating senescence and endoplasmic reticulum stress in primary mouse fibroblasts (41).

In this study, we used primary human dermal fibroblasts to keep the cellular model consistent with the clinical dimension of the *ex vivo* investigation and the previous clinical data. Primary human dermal fibroblasts are key players in regulating skin health. However, additional investigations (Supplementary Figure S3) on human primary keratinocytes may further provide clues on the possible influence of Kera-Diet^®^ on the keratinocytes/fibroblast cross-talks and their positive impact on skin health.

Feathers are a major byproduct of poultry industry. This sustainable and bioavailable source of protein presents bioactive properties. In this study we further provide biological and clinical evidence on the mode of action driving the health benefits of such an active food ingredient on skin metabolism. Furthermore, short peptides found in feather-derived keratin hydrolysates exhibit prebiotic effects promoting ZO-1 and TGF⍺ expression by enterocytes (42) and subsequently improving integrity of the gut barrier *in vivo* (43). Therefore, along with keratinocytes, investigation on other epitheliums may further reveal the potential health benefits of these byproduct-derived bioactives.

## Data Availability

The raw data supporting the conclusions of this article will be made available by the authors, without undue reservation.

## Supplementary material

SUPPLEMENTARY FIGURE S1 Statistical analysis of the absorption profile of circulating amino-acids detected in human serum following Kera-Diet^®^ ingestion. Plots represent the mean ± SD. **p* < 0.05; ***p* < 0.01; *****p* < 0.0001; ns: *p* > 0.05.

SUPPLEMENTARY FIGURE S2 Absorption profile of the 17 amino-acids composing Kera-Diet^®^ and detected in human serum following Kera-Diet^®^ ingestion (*n*=10 volunteers). Plots represent the mean ±SD.

SUPPLEMENTARY FIGURE S3 Primary human keratinocytes subjected to *ex vivo* procedures. Determination of glycosaminoglycans production.

SUPPLEMENTARY TABLE S1 Kera-Diet^®^ amino acid profile.

SUPPLEMENTARY TABLE S2 Transcriptomic analysis in primary human dermal fibroblasts.

## References

[1] Madison KC. Barrier function of the skin: "la raison d'être" of the epidermis. J Invest Dermatol. (2003) 121:231–41. doi: 10.1046/j.1523-1747.2003.12359.x, 12880413

[2] Proksch E Brandner JM Jensen J-M. The skin: an indispensable barrier. Exp Dermatol. (2008) 17:1063–72. doi: 10.1111/j.1600-0625.2008.00786.x, 19043850

[3] Kitada K Nishiyama A. Potential role of the skin in hypertension risk through water conservation. Hypertension. (2024) 81:468–75. doi: 10.1161/HYPERTENSIONAHA.123.20700, 37942635

[4] Kitada K Nishiyama A. Water and sodium metabolisms in kidney-skin crosstalk. Physiology. (2025) 41:2025. doi: 10.1152/physiol.00038.2025 41263403

[5] Legrand M Clark AT Neyra JA Ostermann M. Acute kidney injury in patients with burns. Nat Rev Nephrol. (2024) 20:188–200. doi: 10.1038/s41581-023-00769-y, 37758939 PMC13340345

[6] Fitsiou E Pulido T Campisi J Alimirah F Demaria M. Cellular senescence and the senescence-associated secretory phenotype as drivers of skin Photoaging. J Invest Dermatol. (2021) 141:1119–26. doi: 10.1016/j.jid.2020.09.031, 33349436

[7] Jensen JM Proksch E. The skin's barrier. Giornale Italiano Di Dermatol E Venereol. (2009) 144:689–700. 19907407

[8] Quan T Little E Quan H Qin Z Voorhees JJ Fisher GJ. Elevated matrix metalloproteinases and collagen fragmentation in photodamaged human skin: impact of altered extracellular matrix microenvironment on dermal fibroblast function. J Invest Dermatol. (2013) 133:1362–6. doi: 10.1038/jid.2012.509, 23466932 PMC3637921

[9] Shin J-W Kwon S-H Choi J-Y Na J-I Huh C-H Choi H-R . Molecular mechanisms of dermal aging and antiaging approaches. Int J Mol Sci. (2019) 20:126. doi: 10.3390/ijms20092126, 31036793 PMC6540032

[10] Choi SY Ko EJ Yoo KH Han HS Kim BJ. Effects of hyaluronic acid injected using the mesogun injector with stamp-type microneedle on skin hydration. Dermatol Ther. (2020) 33:e13963. doi: 10.1111/dth.13963, 32621657

[11] Bukhari SNA Roswandi NL Waqas M Habib H Hussain F Khan S . Hyaluronic acid, a promising skin rejuvenating biomedicine: a review of recent updates and pre-clinical and clinical investigations on cosmetic and nutricosmetic effects. Int J Biol Macromol. (2018) 120:1682–95. doi: 10.1016/j.ijbiomac.2018.09.188, 30287361

[12] Gupta D Sharma RR Rashid H Bhat AM Tanveer MA Abdullah ST. Rosmarinic acid alleviates ultraviolet-mediated skin aging via attenuation of mitochondrial and ER stress responses. Exp Dermatol. (2023) 32:799–807. doi: 10.1111/exd.14773, 36811401

[13] Pérez-Sánchez A Barrajón-Catalán E Herranz-López M Micol V. Nutraceuticals for skin care: a comprehensive review of human clinical studies. Nutrients. (2018) 10:403. doi: 10.3390/nu10040403, 29587342 PMC5946188

[14] Elsner P Maibach HI. "Cosmeceuticals: a broad-Spectrum category between cosmetics and drugs". In: Elsner P Maibach HI, editors. Cosmeceuticals and Active Cosmetics, 2nd Edn London: CRC Press (2005)

[15] Solano F. Metabolism and functions of amino acids in the skin. Adv Exp Med Biol. (2020) 1265:187–99. doi: 10.1007/978-3-030-45328-2_11 32761577

[16] Daneault A Prawitt J Fabien Soule V Coxam V Wittrant Y. Biological effect of hydrolyzed collagen on bone metabolism. Crit Rev Food Sci Nutr. (2017) 57:1922–37. doi: 10.1080/10408398.2015.1038377, 25976422

[17] Hou Y Wu Z Dai Z Wang G Wu G. Protein hydrolysates in animal nutrition: industrial production, bioactive peptides, and functional significance. J Anim Sci Biotechnol. (2017) 8:24. doi: 10.1186/s40104-017-0153-9, 28286649 PMC5341468

[18] Le Faouder J Gueho A Lavigne R Wauquier F Boutin-Wittrant L Bouvret E . Human serum, following absorption of fish cartilage hydrolysate, promotes dermal fibroblast healing through anti-inflammatory and immunomodulatory proteins. Biomedicine. (2024) 12:132. doi: 10.3390/biomedicines12092132, 39335645 PMC11430497

[19] Yves H Herman J Uebelhoer M Wauquier F Boutin-Wittrant L Donneau AF . Oral supplementation with fish cartilage hydrolysate in an adult population suffering from knee pain and function discomfort: results from an innovative approach combining an exploratory clinical study and an *ex vivo* clinical investigation. BMC Musculoskelet Disord. (2023) 24:748. doi: 10.1186/s12891-023-06800-4, 37735385 PMC10512646

[20] Wauquier F Boutin-Wittrant L Bouvret E Le Faouder J Roux V Macian N . Benefits of circulating human metabolites from fish cartilage hydrolysate on primary human dermal fibroblasts, an *ex vivo* clinical investigation for skin health applications. Nutrients. (2022) 14:27. doi: 10.3390/nu14235027, 36501057 PMC9737122

[21] Wauquier F Daneault A Granel H Prawitt J Fabien Soule V Berger J . Human enriched serum following hydrolysed collagen absorption modulates bone cell activity: from bedside to bench and vice versa. Nutrients. (2019) 11:249. doi: 10.3390/nu11061249, 31159319 PMC6627680

[22] Tursi F Nobile V Cestone E De Ponti I Lepoudere A Sergheraert R . The effects of an oral supplementation of a natural keratin hydrolysate on skin aging: a randomized, double-blind, placebo-controlled clinical study in healthy women. J Cosmet Dermatol. (2025) 24:e16626. doi: 10.1111/jocd.16626, 39367631 PMC11743286

[23] Monfoulet LE Philippe C Mercier S Coxam V Wittrant Y. Deficiency of G-protein coupled receptor 40, a lipid-activated receptor, heightens in vitro- and in vivo-induced murine osteoarthritis. Exp Biol Med. (2015) 240:854–66. doi: 10.1177/1535370214565078, 25585625 PMC4935402

[24] Poranki D Whitener W Howse S Mesen T Howse E Burnell J . Evaluation of skin regeneration after burns in vivo and rescue of cells after thermal stress in vitro following treatment with a keratin biomaterial. J Biomater Appl. (2014) 29:26–35. doi: 10.1177/0885328213513310, 24272161

[25] Alahyaribeik S Sharifi SD Tabandeh F Honarbakhsh S Ghazanfari S. Stability and cytotoxicity of DPPH inhibitory peptides derived from biodegradation of chicken feather. Protein Expr Purif. (2021) 177:105748. doi: 10.1016/j.pep.2020.105748, 32911063

[26] Pei X-D Li F Gao T-T Su L-Y Yu F-T Shi P . Utilization of feather keratin waste to antioxidant and migration-enhancer peptides by *Bacillus licheniformis* 8-4. J Appl Microbiol. (2023) 134:lxad005. doi: 10.1093/jambio/lxad005 36639131

[27] Olariu L Dumitriu BG Gaidau C Stanca M Tanase LM Ene MD . Bioactive low molecular weight keratin hydrolysates for improving skin wound healing. Polymers. (2022) 14:1125. doi: 10.3390/polym14061125, 35335455 PMC8955321

[28] Feng M Betti M. Transepithelial transport efficiency of bovine collagen hydrolysates in a human Caco-2 cell line model. Food Chem. (2017) 224:242–50. doi: 10.1016/j.foodchem.2016.12.044, 28159262

[29] Lebeaupin C Vallee D Hazari Y Hetz C Chevet E Bailly-Maitre B. Endoplasmic reticulum stress signalling and the pathogenesis of non-alcoholic fatty liver disease. J Hepatol. (2018) 69:927–47. doi: 10.1016/j.jhep.2018.06.008, 29940269

[30] Cullinan SB Diehl JA. PERK-dependent activation of Nrf2 contributes to redox homeostasis and cell survival following endoplasmic reticulum stress. J Biol Chem. (2004) 279:20108–17. doi: 10.1074/jbc.M314219200, 14978030

[31] Cullinan SB Zhang D Hannink M Arvisais E Kaufman RJ Diehl JA. Nrf2 is a direct PERK substrate and effector of PERK-dependent cell survival. Mol Cell Biol. (2003) 23:7198–209. doi: 10.1128/MCB.23.20.7198-7209.2003, 14517290 PMC230321

[32] Jurkin J Henkel T Nielsen AF Minnich M Popow J Kaufmann T . The mammalian tRNA ligase complex mediates splicing of XBP1 mRNA and controls antibody secretion in plasma cells. EMBO J. (2014) 33:2922–36. doi: 10.15252/embj.201490332, 25378478 PMC4282640

[33] Wang M Kaufman RJ. Protein misfolding in the endoplasmic reticulum as a conduit to human disease. Nature. (2016) 529:326–35. doi: 10.1038/nature17041, 26791723

[34] Matos L Gouveia AM Almeida H. ER stress response in human cellular models of senescence. J Gerontol A Biol Sci Med Sci. (2015) 70:924–35. doi: 10.1093/gerona/glu129, 25149687

[35] Valieva Y Ivanova E Fayzullin A Kurkov A Igrunkova A. Senescence-associated beta-galactosidase detection in pathology. Diagnostics. (2022) 12:2309. doi: 10.3390/diagnostics12102309 36291998 PMC9599972

[36] Costa A Pegas Pereira ES Assumpção EC dos Calixto Santos FB Ota FS de Oliveira Pereira M . Assessment of clinical effects and safety of an oral supplement based on marine protein, vitamin C, grape seed extract, zinc, and tomato extract in the improvement of visible signs of skin aging in men. Clin Cosmet Investig Dermatol. (2015) 8:319–28. doi: 10.2147/CCID.S79447 PMC4492544 26170708

[37] Ito N Seki S Ueda F. Effects of composite supplement containing collagen peptide and ornithine on skin conditions and plasma IGF-1 levels—a randomized, double-blind, placebo-controlled trial. Mar Drugs. (2018) 16:482. doi: 10.3390/md16120482, 30513923 PMC6315531

[38] Schunck M Zague V Oesser S Proksch E. Dietary supplementation with specific collagen peptides has a body mass index-dependent beneficial effect on cellulite morphology. J Med Food. (2015) 18:1340–8. doi: 10.1089/jmf.2015.0022, 26561784 PMC4685482

[39] Schwartz SR Hammon KA Gafner A Dahl A Guttman N Fong M . Novel hydrolyzed chicken sternal cartilage extract improves facial epidermis and connective tissue in healthy adult females: a randomized, double-blind, placebo-controlled trial. Altern Ther Health Med. (2019) 25:12–29. 31221944

[40] Schwartz SR Park J. Ingestion of BioCell collagen®, a novel hydrolyzed chicken sternal cartilage extract; enhanced blood microcirculation and reduced facial aging signs. Clin Interv Aging. (2012) 7:267–73. doi: 10.2147/CIA.S32836, 22956862 PMC3426261

[41] Xie W Deng L Qian R Huang X Liu W Tang S. Curculigoside attenuates endoplasmic reticulum stress-induced epithelial cell and fibroblast senescence by regulating the SIRT1-P300 signaling pathway. Antioxidants. (2024) 13:420. doi: 10.3390/antiox13040420, 38671868 PMC11047561

[42] Ke K Sun Y He T Liu W Wen Y Liu S . Effects of feather hydrolysates generated by probiotic *Bacillus licheniformis* WHU on gut microbiota of broiler and common carp. J Microbiol. (2024) 62:–118. doi: 10.1007/s12275-024-00118-z, 38421547

[43] Zhang J Liang M Wu L Yang Y Sun Y Wang Q . Bioconversion of feather waste into bioactive nutrients in water by *Bacillus licheniformis* WHU. Appl Microbiol Biotechnol. (2023) 107:7055–70. doi: 10.1007/s00253-023-12795-8, 37750916

[44] Wauquier F Mevel E Krisa S Richard T Valls J Hornedo-Ortega R . Chondroprotective properties of human-enriched serum following polyphenol extract absorption: results from an exploratory clinical trial. Nutrients. (2019) 11:71. doi: 10.3390/nu11123071, 31888255 PMC6950735

[45] Kleinnijenhuis AJ van Holthoon FL Maathuis AJH Vanhoecke B Prawitt J Wauquier F . Non-targeted and targeted analysis of collagen hydrolysates during the course of digestion and absorption. Anal Bioanal Chem. (2020) 412:973–82. doi: 10.1007/s00216-019-02323-x, 31872275 PMC7005076

[46] Wauquier F Boutin-Wittrant L Viret A Guilhaudis L Oulyadi H Bourafai-Aziez A . Metabolic and anti-inflammatory protective properties of human enriched serum following artichoke leaf extract absorption: results from an innovative *ex vivo* clinical trial. Nutrients. (2021) 13:653. doi: 10.3390/nu13082653, 34444810 PMC8398945

[47] Wauquier F Boutin-Wittrant L Pourtau L Gaudout D Moras B Vignault A . Circulating human serum metabolites derived from the intake of a saffron extract (safr'inside(TM)) protect neurons from oxidative stress: consideration for depressive disorders. Nutrients. (2022) 14:511. doi: 10.3390/nu14071511, 35406124 PMC9002571

[48] Wauquier F Boutin-Wittrant L Krisa S Valls J Langhi C Otero YF . Circulating human metabolites resulting from TOTUM-070 absorption (a plant-based, polyphenol-rich ingredient) improve lipid metabolism in human hepatocytes: lessons from an original *ex vivo* clinical trial. Nutrients. (2023) 15:903. doi: 10.3390/nu15081903, 37111121 PMC10145174

[49] Pourtau L Wauquier F Boutin-Wittrant L Gaudout D Moras B Vignault A . Reduced production of pro-inflammatory and pro-catabolic factors by human serum metabolites derived from a patented saffron extract intake. Pharmaceutics. (2024) 16:336. doi: 10.3390/pharmaceutics16030336, 38543230 PMC10974611

[50] Wauquier F Chavanelle V Bouchard-Mercier A Boutin-Wittrant L Otero YF Krisa S . Bioavailable human metabolites from TOTUM-448 (plant-based formulation) maintain liver cell functionality in a hyperlipidic context that drives MASLD onset. Sci Rep. (2025) 16:556. doi: 10.1038/s41598-025-32556-z, 41402426 PMC12824265

[51] Wauquier F Ripoche D Boutin-Wittrant L Otero YF Krisa S Valls J . TOTUM-854 human circulating bioactives preserve endothelial cell function. Nutrients. (2025) 17:331. doi: 10.3390/nu17081331, 40284196 PMC12030166

