# Supplementary figures and images for: Bioavailable human metabolites of a keratin-derived hydrolysate promote primary human dermal fibroblast activities and protect against oxidative stress-related damages
Source: Front Nutr. 2026 May 14;13:1812320. doi: 10.3389/fnut.2026.1812320 (PMC13218331; doi:10.3389/fnut.2026.1812320)

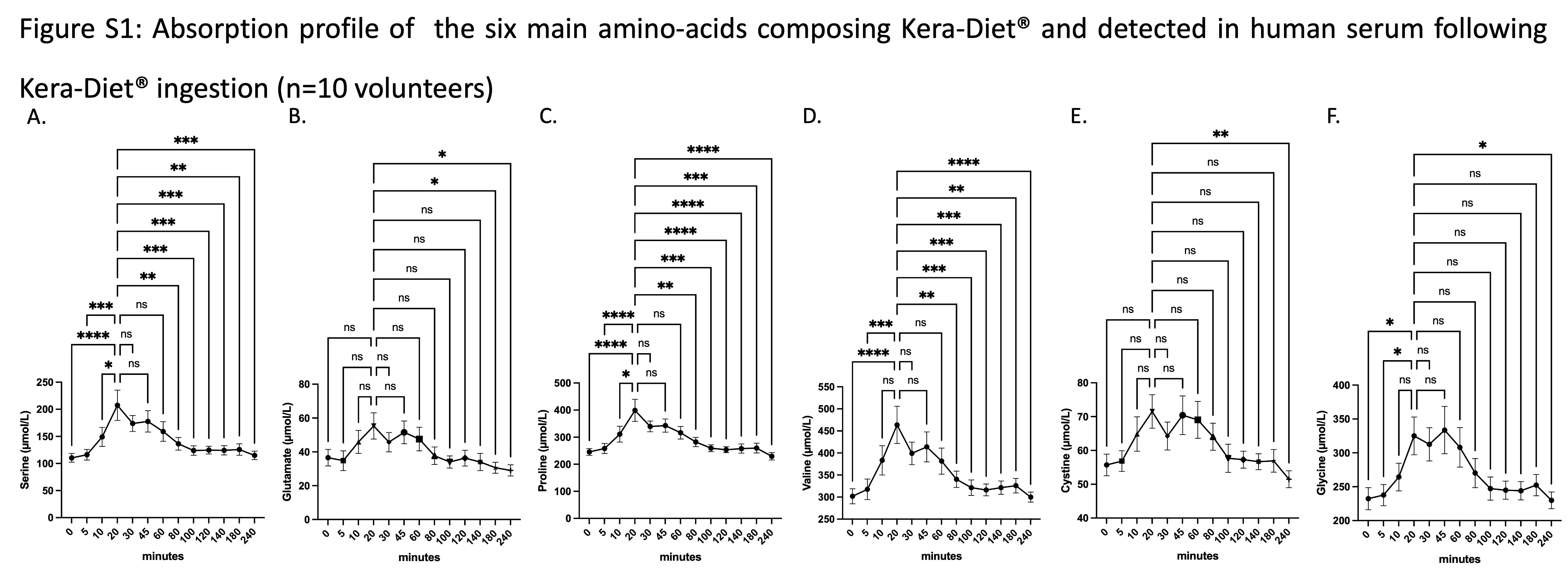

Supplement: SUPPLEMENTARY FIGURE S1 — Statistical analysis of the absorption profile of circulating amino-acids detected in human serum following Kera-Diet® ingestion. Plots represent the mean ± SD. *p < 0.05; **p < 0.01; ****p < 0.0001; ns: p > 0.05. [file Supplementary_file_1.jpg]

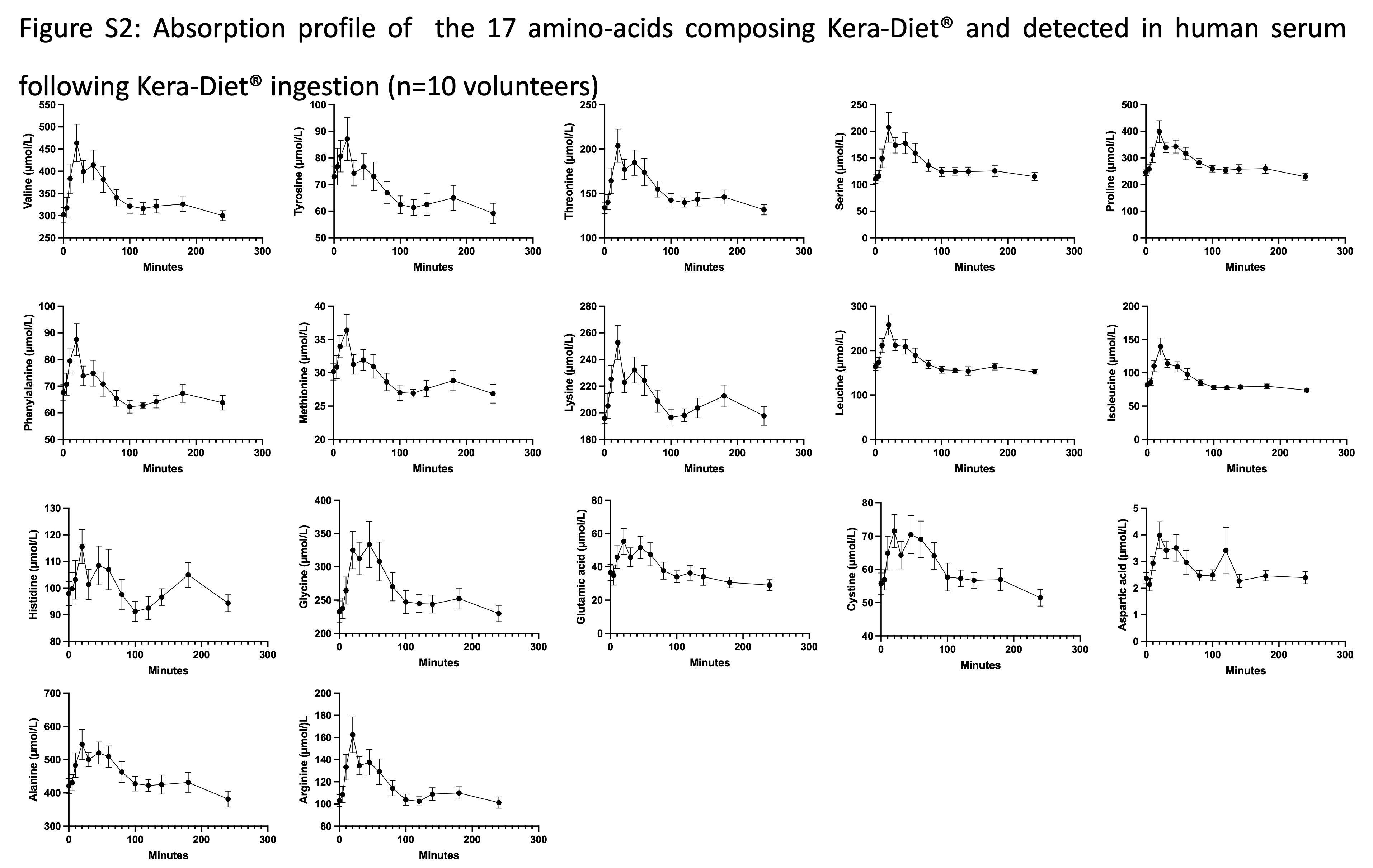

Supplement: SUPPLEMENTARY FIGURE S2 — Absorption profile of the 17 amino-acids composing Kera-Diet® and detected in human serum following Kera-Diet® ingestion (n=10 volunteers). Plots represent the mean ±SD. [file Supplementary_file_2.jpg]

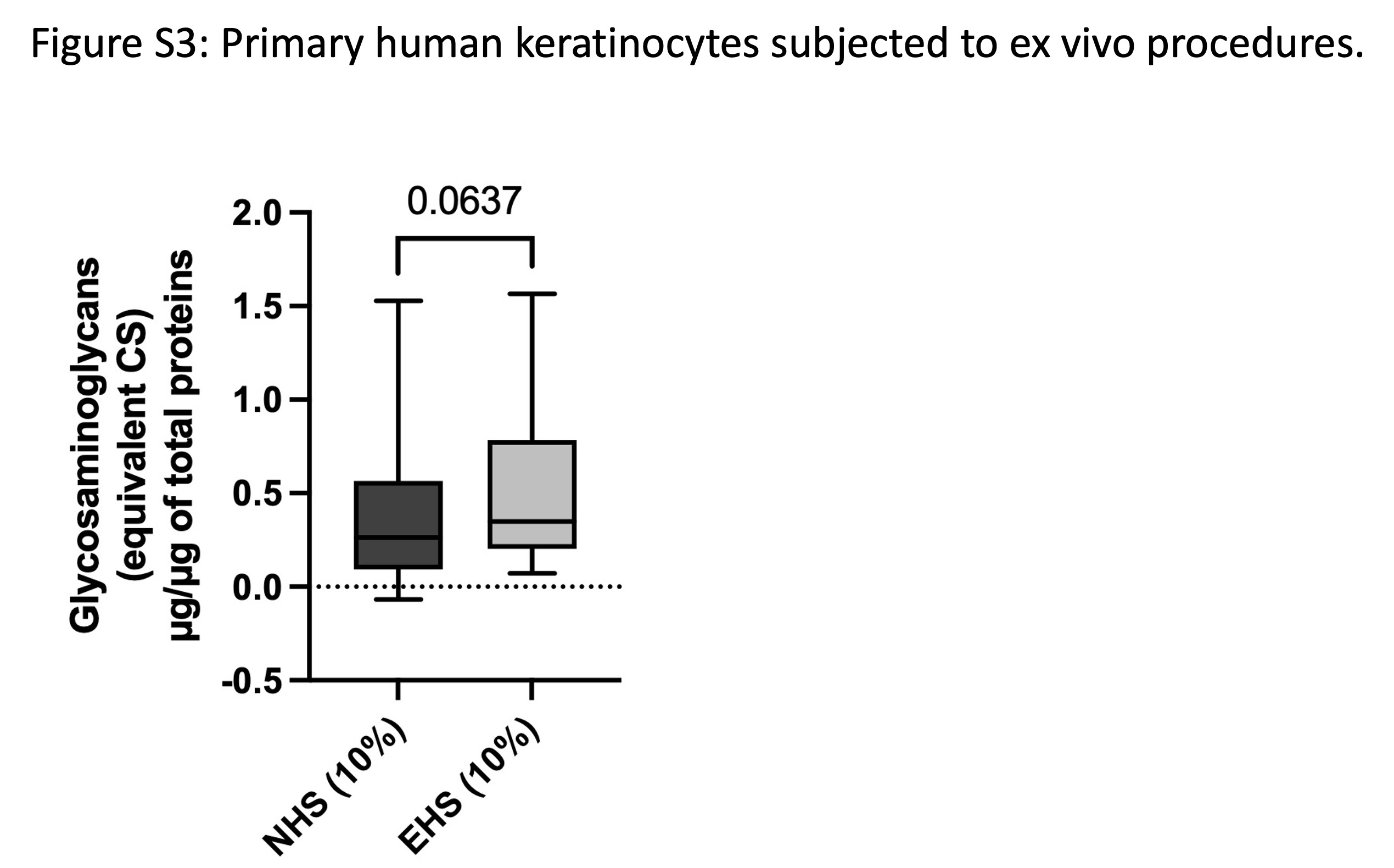

Supplement: SUPPLEMENTARY FIGURE S3 — Primary human keratinocytes subjected to ex vivo procedures. Determination of glycosaminoglycans production. [file Supplementary_file_3.jpg]

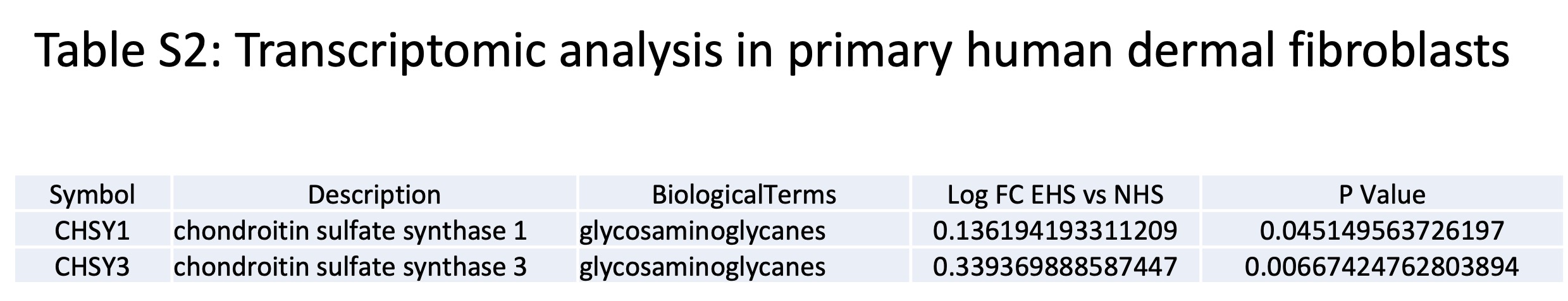

Supplement: SUPPLEMENTARY TABLE S1 — Kera-Diet® amino acid profile. [file Supplementary_file_4.jpg]
