# Supplementary material for: Bioavailable human metabolites of a keratin-derived hydrolysate promote primary human dermal fibroblast activities and protect against oxidative stress-related damages
Source: Front Nutr. 2026 May 14;13:1812320. doi: 10.3389/fnut.2026.1812320 (PMC13218331; doi:10.3389/fnut.2026.1812320)
Supplement: SUPPLEMENTARY TABLE S2 — Transcriptomic analysis in primary human dermal fibroblasts. [file Supplementary_file_5.pptx]

## Slide 1
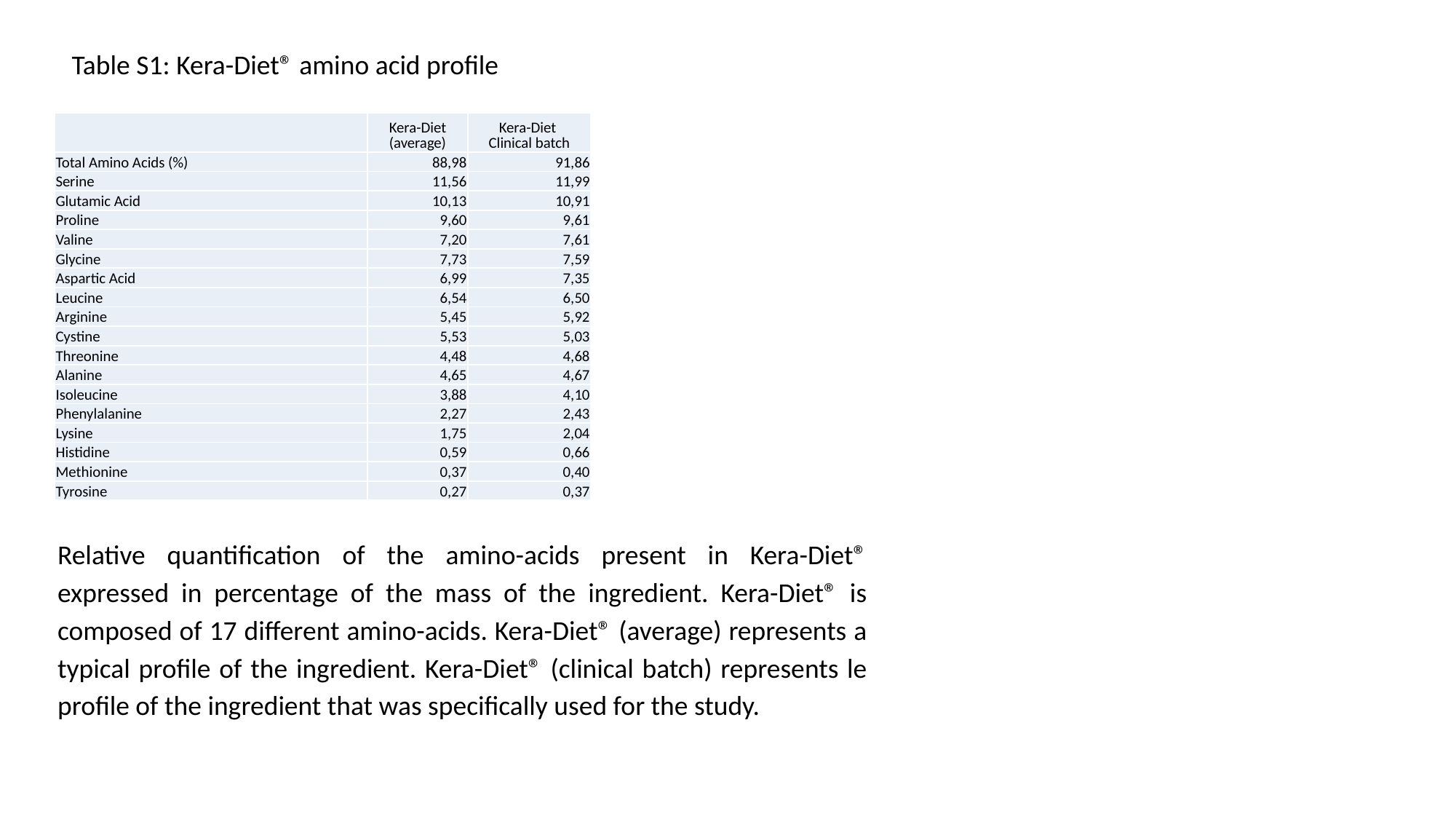

Table S1: Kera-Diet® amino acid profile
| | Kera-Diet (average) | Kera-Diet Clinical batch |
| --- | --- | --- |
| Total Amino Acids (%) | 88,98 | 91,86 |
| Serine | 11,56 | 11,99 |
| Glutamic Acid | 10,13 | 10,91 |
| Proline | 9,60 | 9,61 |
| Valine | 7,20 | 7,61 |
| Glycine | 7,73 | 7,59 |
| Aspartic Acid | 6,99 | 7,35 |
| Leucine | 6,54 | 6,50 |
| Arginine | 5,45 | 5,92 |
| Cystine | 5,53 | 5,03 |
| Threonine | 4,48 | 4,68 |
| Alanine | 4,65 | 4,67 |
| Isoleucine | 3,88 | 4,10 |
| Phenylalanine | 2,27 | 2,43 |
| Lysine | 1,75 | 2,04 |
| Histidine | 0,59 | 0,66 |
| Methionine | 0,37 | 0,40 |
| Tyrosine | 0,27 | 0,37 |
Relative quantification of the amino-acids present in Kera-Diet® expressed in percentage of the mass of the ingredient. Kera-Diet® is composed of 17 different amino-acids. Kera-Diet® (average) represents a typical profile of the ingredient. Kera-Diet® (clinical batch) represents le profile of the ingredient that was specifically used for the study.
